# Microscope studies of symptomless growth of *Botrytis cinerea* in *Lactuca sativa* and *Arabidopsis thaliana*

**DOI:** 10.1111/ppa.13683

**Published:** 2022-12-08

**Authors:** Christy J. Emmanuel, Henk‐Jan Schoonbeek, Michael W. Shaw

**Affiliations:** ^1^ Department of Botany, Faculty of Science University of Jaffna Jaffna Sri Lanka; ^2^ John Innes Centre, Norwich Research Park Norwich UK; ^3^ School of Agriculture, Policy and Development University of Reading Reading UK

**Keywords:** confocal microscopy, endophyte, grey mould, grey mould, latent infection, lettuce

## Abstract

The grey mould pathogen *Botrytis cinerea* forms systemic associations in some hosts, spreading into plant organs produced a considerable time after initial infection. These infections may have no macroscopic symptoms during much of the hosts' lifetime and are at least partially within the host tissue. The aim of the studies reported here was to locate and visualize these infections at a cellular level in *Lactuca sativa* (lettuce) and *Arabidopsis thaliana*. Symptomless but infected plants were produced by dry spore inoculation of plants growing in conditions previously shown to result in fungal spread from the initial inoculation site to newly developing plant organs. Tissue taken from inoculated plants was examined using confocal laser scanning microscopy. Two *B*. *cinerea* isolates were used: B05.10 and its GFP‐labelled derivative Bcgfp1‐3. Spore germination on leaf surfaces was followed by development of subcuticular inclusions and plant cell damage in single infected epidermal cells and sometimes a few nearby cells. Sparsely branched long hyphae arose and spread from the inclusions, mostly on the outer surface of the epidermal layer but occasionally below the cuticle or epidermal cells, where further inclusions formed. This was consistent with the pattern in time of recovery of *B*. *cinerea* from surface‐sterilized leaf tissue. In the late symptomless phase, mycelium arising from internal fungal inclusions formed mycelial networks on the surface of leaves. Symptomless exterior mycelium grew on the roots in *A*. *thaliana*.

## Graphical abstract

Fluorescence microscopy shows that the grey mould fungus *Botrytis cinerea* can spread without symptoms on lettuce and *Arabidopsis* by alternating subcuticular and external hyphal growth.

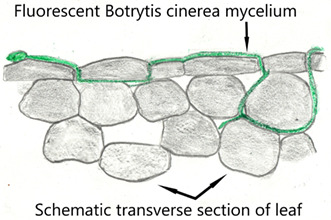

## 1 INTRODUCTION

The fungus *Botrytis cinerea* is well known as a necrotroph, but it also shows latent or quiescent infection in some host tissues (van Kan et al., 2014; Shaw et al., 2016). In quiescent infections, the pathogen ceases to grow and is restricted to one or a few cells until the host tissue becomes more widely favourable to growth of the pathogen, typically as sugar concentrations rise on ripening. By contrast, in other hosts or conditions, *Botrytis* species can spread into new organs as they grow and develop, without macroscopic symptoms. In such hosts, spreading necrotic symptoms typically develop under conducive conditions at or during flowering or fruit ripening (Barnes & Shaw, 2003). *B*. *cinerea* has been shown to cause this type of symptomless systemic infection in, among others, lettuce (*Lactuca sativa*), primula (*Primula* × *polyantha* and *P*. *vulgaris*), *Arabidopsis thaliana* and dandelion (*Taraxacum* agg.) (Emmanuel et al., 2018; Rajaguru & Shaw, 2010; Shaw et al., 2016; Sowley et al., 2010). Such infection has been detected by isolating live fungus from symptomless tissue and by detection of scarce RNA species typical of the pathogen (Emmanuel et al., 2018). It would be desirable to understand better the movement of the pathogen within host tissues by direct observation.

Several previous microscope studies have been done to elucidate the stages in necrotrophic or quiescent infection of *B*. *cinerea*. Williamson and Duncan (1989) studied the infection of raspberry flowers by *B*. *cinerea* using scanning electron microscopy. *B*. *cinerea* infection of grape flowers was also monitored under electron microscopy by Viret et al. (2004). All such studies suffer from the need to use very concentrated inocula because of the limited areas that can be scanned and to avoid possible confusion with other fungi. The infection process of *B*. *cinerea* on rose petals was studied by Williamson et al. (1995) using fluorescence microscopy. However, this was a directly necrotrophic infection that is likely to differ in detail, even in the early stages, from a systemic symptomless infection. Sowley et al. (2010) used the monoclonal antibody BC‐KH4 (Bossi & Dewey, 1992) to label *B*. *cinerea* for staining with anti‐mouse IgG Texas Red in lettuce. Hyphae were seen inside symptomless root and leaf tissue (Sowley et al., 2010). However, the antibody BC‐KH4 binds to an excreted carbohydrate, which may not be present at all stages of growth and may diffuse from the mycelium or remain after the death of a fungal cell; the antibody is also not isolate specific.

There have been several attempts to trace the systemic infection process in host plants using green fluorescent protein (GFP)‐transformed *B*. *cinerea*. Li et al. (2007) successfully inserted the *GFP* gene into *B*. *cinerea*. The gene was expressed in hyphae and conidia both in vitro and in planta, albeit weakly, without showing differences in morphology or pathogenicity of the fungus. Rajaguru (2008) examined *P.* × *polyantha* inoculated with conidia from a culture of *B*. *cinerea* isolated from surface‐sterilized, symptomless parts of the plants and transformed with GFP. The GFP‐expressing pathogen could not be visualized inside leaves or other plant tissues until they showed symptoms. This result is hard to interpret unambiguously for several reasons: *P.* × *polyantha* leaves are much thicker than the depth of field of the confocal microscope used, so the interior of the leaf and vascular system could not be examined; green autofluorescence from plant tissues interfered with the GFP signal; and GFP was weakly expressed in the transformant used. Leroch et al. (2011) created an improved GFP‐marked tool using a codon‐optimized intron‐containing gene (*bcgfp1*) coding for eGFP.

The objective of the study reported here was to supplement molecular and culture‐based results concerning symptomless systemic infection by *B*. *cinerea* by direct observation using confocal microscopy. We chose *L*. *sativa* because it has been used extensively in studies of systemic infections (Emmanuel et al., 2018; Shaw et al., 2016; Sowley et al., 2010). Comparisons were made with *A. thaliana* because of its extensive role as a model species in plant sciences. Earlier experiments verified that the conditions used led to internal systemic infection in both species; however, the experiments reported in Emmanuel et al. (2018) suggested differences in gene activation during infection in the two host species. This may reflect differences in the way in which *B*. *cinerea* grows within and on *L*. *sativa* and *A*. *thaliana*.

## 2 MATERIALS AND METHODS

### 2.1 Fungal isolates

*B*. *cinerea* isolate B05.10 and its GFP‐labelled derivative Bcgfp1‐3 were used. Good quality, annotated sequence of the isolate has been published (van Kan et al., 2017) and the isolate has been extensively used for physiological study (Corwin et al., 2016; van Kan, 2005; Leroch et al., 2011; Stefanato et al., 2009). In conventional inoculations, with concentrated suspensions applied in droplets to detached leaves held at 100% humidity, both isolates are pathogenic on lettuce, *A*. *thaliana* and numerous other hosts (Caseys et al., 2021; Shaw et al., 2016; Stefanato et al., 2009). A single spore isolate, Bcgfp1‐3, from the GFP‐transformed culture Bcgfp1, made in the Department of Biology, University of Kaiserslautern, Kaiserslautern, Germany (Leroch et al., 2011), was selected to generate inoculum for fluorescence microscopy. It had moderate virulence on *A*. *thaliana* Col‐0 plants after droplet inoculation with concentrated spore suspensions in nutrient solution.

### 2.2 Host plants

Lettuce (*Lactuca sativa*) cv. Tom Thumb (Thomson & Morgan) seed, not fungicide treated, was used in all experiments on lettuce that are reported here. Surface‐disinfested seeds were sown one per cell in F1 Seed & Modular compost (Levington) in multicell seedling trays, covered with a thin layer of compost. The compost was kept damp to touch at all times. After 4 weeks, the seedlings were transplanted into 1 L pots filled with potting compost (John Innes 2 compost + 4 g/L Osmocote). For the first 2 weeks after sowing, pots were watered from below every day sufficient to keep the compost just moist, and then were watered at 2‐day intervals.

*A. thaliana* (Col‐0) seeds were surface disinfested in 70% ethanol for 2 min and then in 20% domestic bleach (Domestos, Unilever: 5% NaOCl in alkaline solution with surfactants) for 5 min and finally thoroughly rinsed in sterile water five or six times. After surface disinfestation seeds were stratified at 4°C for 4 days. Seeds were sown singly on the surface of Clover Seed and Modular Compost (Clover) in pots covered with transparent polystyrene propagation covers with vents and grown on in these propagators. Pots were watered as for lettuce.

In experiments with Bcgfp1‐3, plants of both species were held in containment conditions within a controlled environment cabinet at 65% relative humidity with a 16 h light period at 22°C and an 8 h dark period at 18°C. The light intensity was 80–85 μmol/m^2^/s, with a daily light integral of about 4.6 mol/m^2^. This is equivalent to deep shade: about 10%–15% of naturally experienced outdoor values (Poorter et al., 2016) in spring in temperate regions, when both species germinate and grow. This was the maximum intensity attainable within the containment growth chamber.

### 2.3 Fungus cultivation and inoculation

For inoculation, spores were collected from 3‐week‐old cultures growing on malt extract agar plates, by tapping the plates on a piece of aluminium foil inside the flow cabinet. Five milligrams of spores were serially diluted in talc powder through five stages, each by a factor of 10. For both host species, this diluted spore dust was dispersed into the air above the plants and allowed to settle. Controls were dusted with talc powder only.

Lettuce inoculation was performed at the two‐true‐leaf stage in entire seed trays that were immediately covered with transparent lids for 2 days to provide higher humidity.

*A*. *thaliana* plants growing in a propagator were inoculated using a porthole in the top of the cover, which was about 13 cm above the plants and otherwise kept covered with sticky tape. During each inoculation, clean coverslips were placed among the plants and the spore deposition per unit area was measured by microscope counts.

### 2.4 Sampling

**FIGURE 1:**
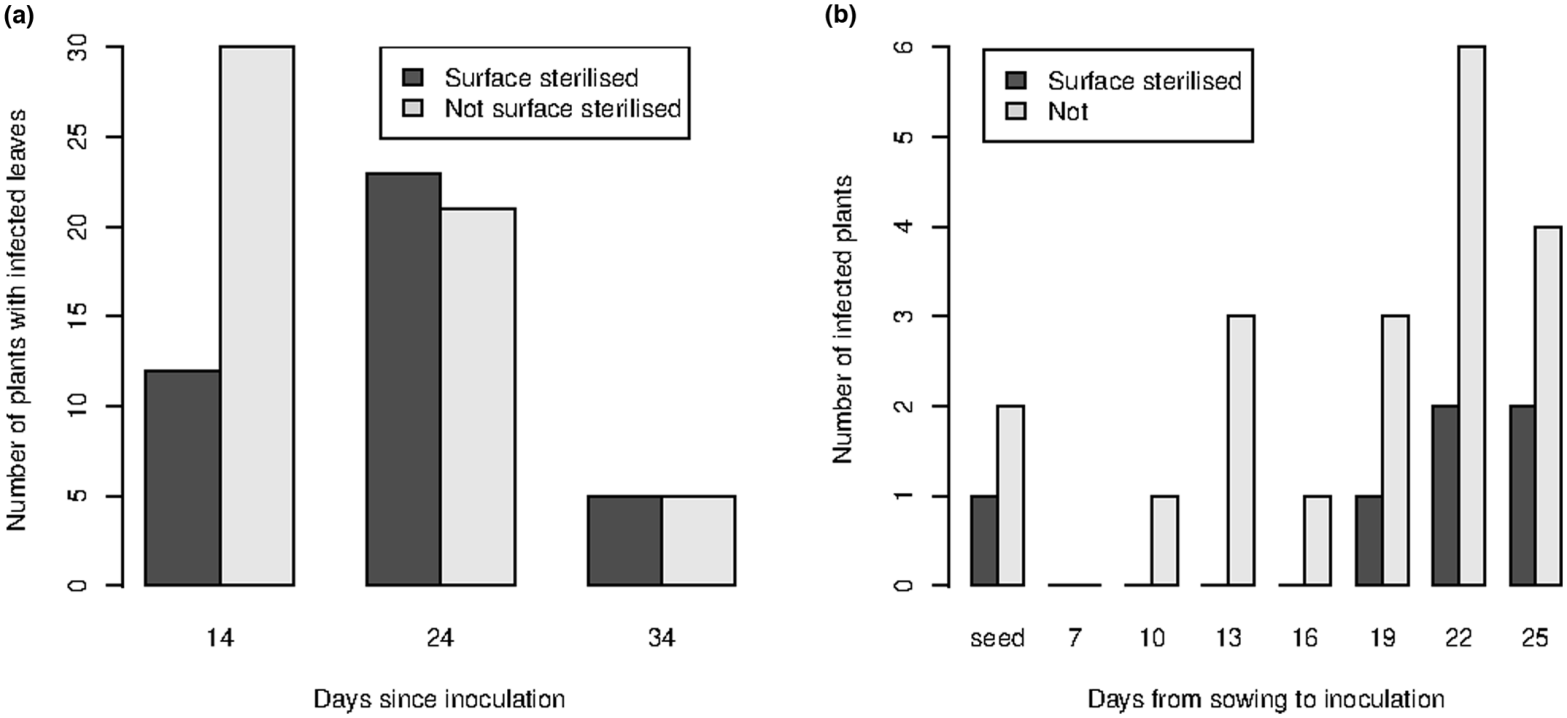
(a) Recovery of *Botrytis cinerea* from the three most recently expanded leaves of *Lactuca sativa* at successive times after inoculation. (Summarized data from Emmanuel et al., 2018.) Before plating on *Botrytis* selective medium, leaves were either rinsed in deionized water (“not disinfested”) or immersed in ethanol and then bleach (“disinfested”). *n* = 30 plants on each sampling occasion. (b) Recovery of *B*. *cinerea* from surface disinfested or water‐rinsed rosette leaves of *Arabidopsis thaliana* at inflorescence development (35 days after sowing) following dry inoculation at successive time‐points after sowing. Seed inoculated plants are labelled “seed”. *n* = 6 plants on each sampling occasion.

Choice of sampling times in experiments with Bcgfp1‐3 was based on earlier experiments in greenhouse conditions using unmodified B05.10 cultures (Figure 1a; Emmanuel et al., 2018). These results were used to choose the period over which microscope observations of GFP‐modified *B*. *cinerea* Bcgfp1‐3 were made.

#### 2.4.1 L. sativa

Seedlings at the two‐true‐leaf stage were dust inoculated with B05.10 as described in the previous section. The first samples were taken 2 days after inoculation; in each sample, five seedlings were uprooted, cleaned in running water and blotted dry. Plants were transplanted at 7 days postinoculation (dpi) and a further three samplings of symptomless tissues were performed at 14, 24 and 34 dpi. In each of these samplings, 10 apparently healthy plants were uprooted and leaves were collected as pairs. Leaf samples were also collected from control plants. Roots were not sampled. To disinfest samples, they were immersed in 70% ethanol for 1 min, then in a 50% solution of bleach (Domestos, Unilever as above) for 1 min and finally rinsed three times in sterile distilled water. From each leaf a 1.5 × 1.5 cm section was taken, placed on *Botrytis* selective medium (BSM) (Edwards & Seddon, 2001), and observed at intervals of a few days with a dissection microscope for characteristic sporulating structures and browning of the medium. The recovery on BSM from disinfested or undisinfested samples is attributable to systemicity arising from growth inside the leaves or on the external surface of the plant respectively (Shaw et al., 2016; Figure 1).

#### 2.4.2 A. thaliana

Inoculations with B05.10 were carried out on seed and at the rosette growth stage at 7, 10, 13, 16, 19, 22 and 25 days after sowing with a final sampling date at 35 days after sowing, by which time plants had erect flowering structures. Samples of tissues from rosette leaves, stem leaves and stems of inoculated *A*. *thaliana* were collected at successive time points 3–4 days apart, starting from 2 days after inoculation. Sample plants were uprooted, then dissected into aerial and root portions. The root samples were washed in running water to remove compost and other debris. From each plant, two samples were taken for each of two stem segments (c.2 cm), three rosette leaves, two stem leaves, a piece of root (c.2 cm long) and two inflorescences. One of each pair of samples was surface disinfested and one not. A subsample of each tissue type treated in each way was plated on BSM and examined at intervals as for lettuce.

PCR was used to quantify *B*. *cinerea* in 40 ng aliquots of *A*. *thaliana* DNA extracted from stem or stem–leaf tissue samples taken 35 days after sowing from plants inoculated at 0 (i.e., seed), 10, 16 and 22 days after sowing. Samples were ground in liquid nitrogen and stored at −20°C where necessary. DNA was extracted using Plant Mini Kits (QIAGEN) according to the manufacturer's instructions. Extracts were adjusted to 10 ng/μl. For quantitative PCR (qPCR), 4 μl of DNA extract were mixed with 10 μl of SYBR Green JumpStart Ready Mix (Sigma‐Aldrich) and 0.6 μl of 300 nM primers 5′‐GCTGTAATTTCAATGTGCAGAATCC‐3′ and 5′‐GGAGCAACAATTAATCGCATTTC‐3′ (amplifying the species‐specific rDNA intergenic spacer region; Suarez et al., 2005), for *B*. *cinerea* quantification, or 5′‐CTTATCGGATTTCTCTATGTTTGGC‐3′ and 5′‐GAGCTCCTGTTTATTTAACTTGTACATACC‐3′ (amplifying α shaggy kinase DNA; Gachon & Saindrenan, 2004) for host standardization. Amplifications were run in duplicate using a Rotorgene 6000 (Corbett Life Sciences) with a 10 min initial melt at 95°C followed by 40 cycles of 60°C for 1 min and 95°C for 15 s. After each run melting curves were acquired between 60°C and 95°C and checked. For quantification, standard curves were prepared based on five 10‐fold serial dilutions of 2.5 ng/μl *B*. *cinerea* B05.10 DNA from cultured spores or 20 ng/μl *A*. *thaliana* Col‐1 DNA from stems (with stem–leaves).

### 2.5 Microscopy

Lettuce plants were inoculated at the two‐true‐leaf stage. *Arabidopsis* plants were inoculated at about 21 days after seed sowing, while the plants were at the rosette growth stage. Three methods were then used to visualize *B*. *cinerea* in host tissue.

First and most directly, tissue samples from plants that had been dry inoculated with Bcgfp1‐3 were mounted in water and immediately observed by conventional fluorescence microscopy under blue light (495 nm), looking for green fluorescence at 509 nm. Slides of interest were examined further with a confocal laser scanning microscope (TCS SP5ii; Leica) without further preparation.

Second, some tissue samples from *A*. *thaliana* inoculated with B05.10 were stained with calcofluor white (Sigma‐Aldrich) by vacuum infiltration. A stock stain solution was prepared with a 5 g/L concentration of calcofluor white in sterilized water and the pH adjusted to 10–11 by adding 1 M NaOH. Tissue samples were stained by vacuum with a 100× dilution of this stock solution in water, then mounted on a slide. Initial searches were made with a conventional fluorescent microscope using a blue channel (excitation 405 nm, emission 500 nm) to detect calcofluor white‐stained *B*. *cinerea* and plant cell walls. Slides of interest were selected for observation with a confocal laser scanning microscope.

Third, samples were fixed in a mixture of ethanol and acetic acid (3:1 vol/vol), washed in phosphate buffer (pH 6.8), and then kept in 10% KOH for 2 h before washing in phosphate buffer and staining for 1 h with wheat germ agglutinin‐linked fluorescein isothiocyanate (WGA‐FITC; Sigma) diluted 500× in phosphate buffer (pH 6.8). Samples were then washed in phosphate buffer and counterstained by suspension in propidium iodide solution (10 μg/ml; Sigma) for a further 1 h. After a further wash in phosphate buffer, samples were mounted on a slide in citifluor (Agar scientific, http://www.agarscientific.com) to reduce background fluorescence. Slides were observed under a TCS SP5ii confocal laser scanning microscope, using the software LAS AF (v. 2.7.3.9723). WGA‐FITC‐stained *B*. *cinerea* and sometimes chloroplasts were detected in a green channel (excitation 488 nm, emission 514 nm). Propidium iodide‐stained plant tissues were observed in a red channel (excitation 488 nm, emission 617 nm).

In all cases the transmitted light, bright field image was observed using white light. The bitmap graphic files (Zeiss CLSM format) were processed with the Fiji application (http://fiji.sc/Fiji). Image channels were merged and brightness and contrast were adjusted. *Z* stacks were superimposed to produce 2D projections and 3D images.

### 2.6 Statistical methods

The effect of surface disinfestation and inoculation date in both hosts on the probability of isolating *B*. *cinerea* was analysed by a generalized linear model using a binomial error distribution with logit link function. The differences between dates were tested against χ^2^. If these were significant, the deviance between sterilization treatments was tested against the *F* distribution using the between date deviance as the denominator. Observations of log DNA concentration were analysed using conventional analysis of variance (ANOVA); residuals on the log scale conformed to normality.

At inoculation, approximate estimates of spore density on coverslips placed on the pots were made. Two days after inoculation 10 counts were made of germinated Bcgfp1‐3 spores seen in transects across inoculated leaves of *L*. *sativa* under fluorescent illumination. Otherwise, specific microscope observations were qualitative: fungal mycelium was sparse and no exact numerical comparisons were possible between times or host species.

## 3 RESULTS

### 3.1 Time‐course of infection

Detailed results for *L. sativa* are published in table 1 of Emmanuel et al. (2018). Summarizing the data (Figure 1a) and re‐analysis showed clear differences between times after inoculation (*p* < 0.001) and lesser differences between disinfestation treatments (*p* = 0.02). At 14 days postinoculation (dpi), *B*. *cinerea* was recovered from 30/30 samples of leaves that were not disinfested before plating on BSM, and from12/30 surface‐disinfested leaves. At 24 dpi, when the leaves sampled had not been present at inoculation, *B*. *cinerea* was recovered from 21/30 and 23/30 of disinfested and undisinfested samples, respectively, and at 34 dpi, 5/30 and 5/30 respectively. Therefore, material subsequently grown for microscopy was sampled at intervals between 2 and 30 dpi.

In *A. thaliana*, leaves were sampled at 35 days after sowing. *B*. *cinerea* was recovered from at least one surface‐disinfested tissue sample from plants inoculated as either as seed or 19–25 days after sowing (*p* = 0.008 for differences between inoculation dates, *F* test using the interaction χ^2^ between inoculation date and sterilization treatment as denominator; Figure 1b). The pattern of recovery was similar for undisinfested tissues, but the frequency was much greater (overall recovery from 20/48 plants compared to 6/48 from disinfested plants; *p* < 0.003, *F* test). There was no evidence for the difference in recovery rate due to surface disinfestation differing between times following inoculation (*p* = 0.7, χ^2^ test). Including all time periods, *B*. *cinerea* was not recovered from roots of surface‐disinfested samples but was recovered from roots of 2/48 samples not disinfested, one each on the two last sampling occasions. The temporal pattern was quite different from *L*. *sativa* and the proportion of tissue samples from which *B*. *cinerea* could be recovered was much smaller.

*B*. *cinerea* DNA was detected in aliquots of *A*. *thaliana* DNA from stem or stem–leaf tissue sampled at 35 days after sowing in plants inoculated as seed or at 10, 16 or 22 days after sowing. There was approximately 10× more *B*. *cinerea* in the samples inoculated at 22 days than in earlier ones (*p* < 0.001) (data not shown). Therefore, to maximize the chances of finding *B*. *cinerea* in subsequent tissue samples we chose 22 days after sowing as the inoculation time for dry leaf inoculations of *A*. *thaliana*.

### 3.2 Microscope visualization

#### 3.2.1 *L. sativa* ‘Tom Thumb’

All the images shown were obtained with leaf tissue from plants that were symptomless but had been inoculated.

**FIGURE 2:**
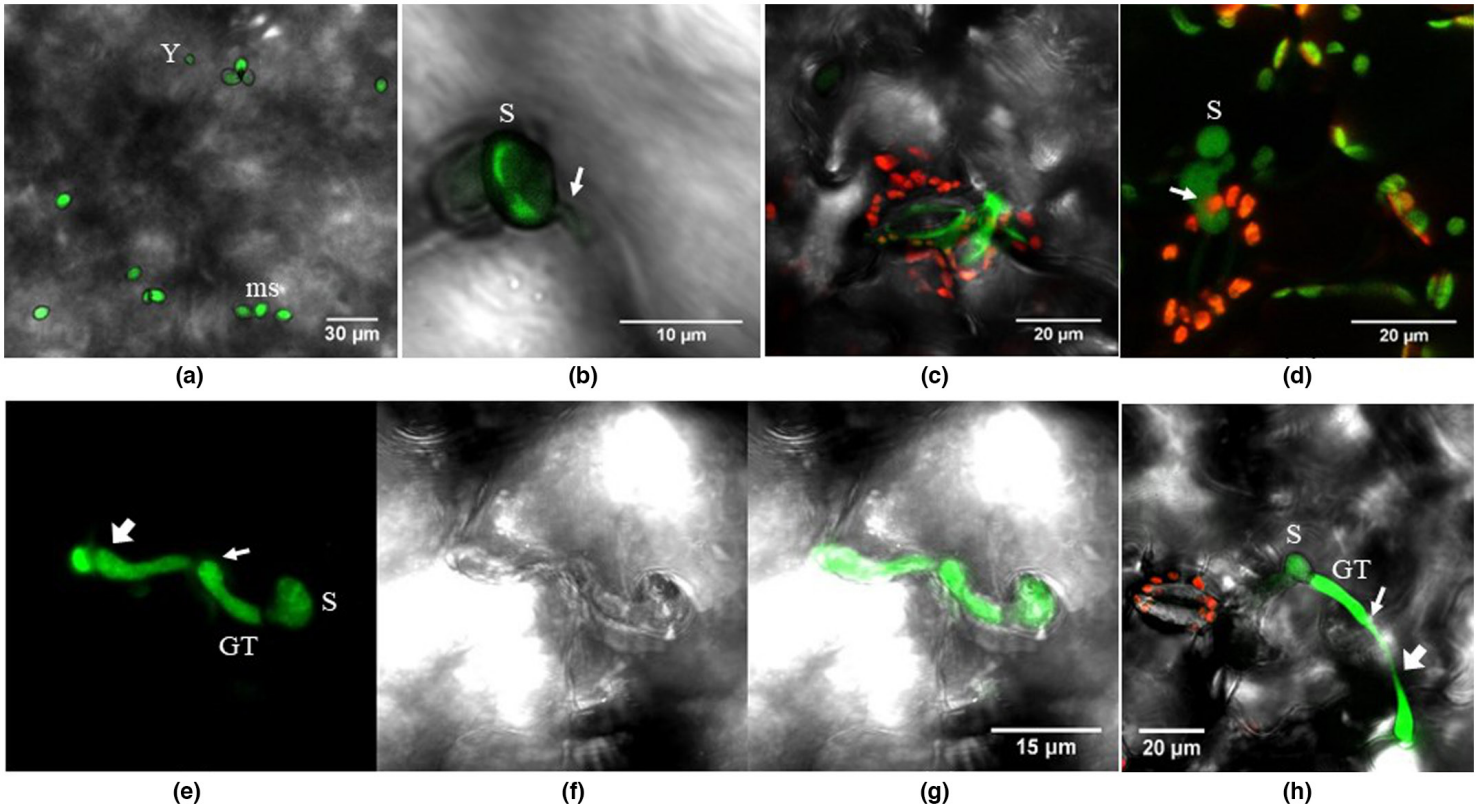
Lettuce leaf up to 10 days after inoculation with GFP‐labelled *Botrytis cinerea* Bcgfp1‐3, viewed by confocal scanning laser microscopy. (a and b) Two days after inoculation, superimposed images from green and bright‐field, with (b) a higher magnification of (a), showing a short germ tube, which penetrates the cuticle layer immediately below the conidium at an epidermal cell junction; conidia (S); approximately spherical microspores (ms). (c) Four days after inoculation, superimposed images from the green and red channels and bright‐field show a germinated conidium has produced a short germ tube very close to the guard cell. (d) Five days after inoculation, superimposed images from the green and red channels show the germ tube of a spore has penetrated a side wall of a guard cell and the tip of the germ tube remains inside the leaf, below the epidermal chloroplasts (arrow). (e–g) Five days after inoculation, images from the green channel (e), bright‐field (f), and superimposed green and bright‐field (g) showing a conidium (S) has germinated on the epidermal cell surface, the germ tube (GT) has grown on the outside of the cuticle until a cell junction, then penetrated the cuticle at the cell junction and grown parallel to the leaf surface below the cuticle (between the two arrows); the hypha has grown out through the cuticle at a junction of three epidermal cells. (h) Ten days after inoculation, superimposed images from the green and red channels and bright field, showing a germ tube (GT) has penetrated the cuticle (thinner arrow), grown parallel to the leaf surface between two epidermal cells, and then re‐emerged onto the leaf surface (broader arrow).

In both host species, development of mycelium from germinated spores was not synchronous or uniform. On inoculated leaves, there were two kinds of nonmycelial structure. The ovoid‐shaped conidia (Figure 2a) were 10.1 ± 1.5 μm long (*n* = 20) and 6.2 ± 0.8 μm wide (*n* = 20), as expected for *B*. *cinerea*; nongerminated microspores with average diameter of 3.2 ± 0.7 μm (*n* = 20) were also seen on leaves present at the time of inoculation (Figure 2a). The density of *germinated* conidia was about three spores/mm^2^ of leaf surface, based on microscope transects across sampled leaves on the first sampling date, 2 days after inoculation. The density of *inoculated* spores was 90 ± 10 spores/mm^2^ (*n* = 10), based on coverslip samples immediately after inoculation. Development of mycelium from spores that germinated, in most cases, stopped after production of short germ tubes, and typically only about five spores per 2 cm^2^ leaf sample (=0.025 spores/mm^2^) showed continued fungal development. Spore counts on leaf surfaces are probably lower than on coverslips because it is unlikely that all spores adhere sufficiently strongly to remain in place during manipulation of plant material for microscopy. Successful spore germination and further development was found all over the leaf epidermis, including on or near to guard cells and intercellular junctions. There were no abnormal structural changes or visible macroscopic or microscopic plant responses in the plant cells adjacent to spores whose development had ceased after germination.

##### Germination

Germinated conidia mostly produced single germ tubes, but occasionally more. The lengths of germ tubes and their mode of penetration were not uniform; some conidia produced a short germ tube and immediately penetrated the epidermal cell wall or guard cell wall (Figure 2b–d); some other conidia produced slightly longer germ tubes that grew parallel to the leaf surface, either completely below the cuticle or intermittently exposed to the outer surface (Figure 2e–g). Germ tube growth was also noted at the cell junctions where the germ tube ran parallel to the cell junction below the cuticle layer (Figure 2e–h).

##### 5 dpi

**FIGURE 3:**
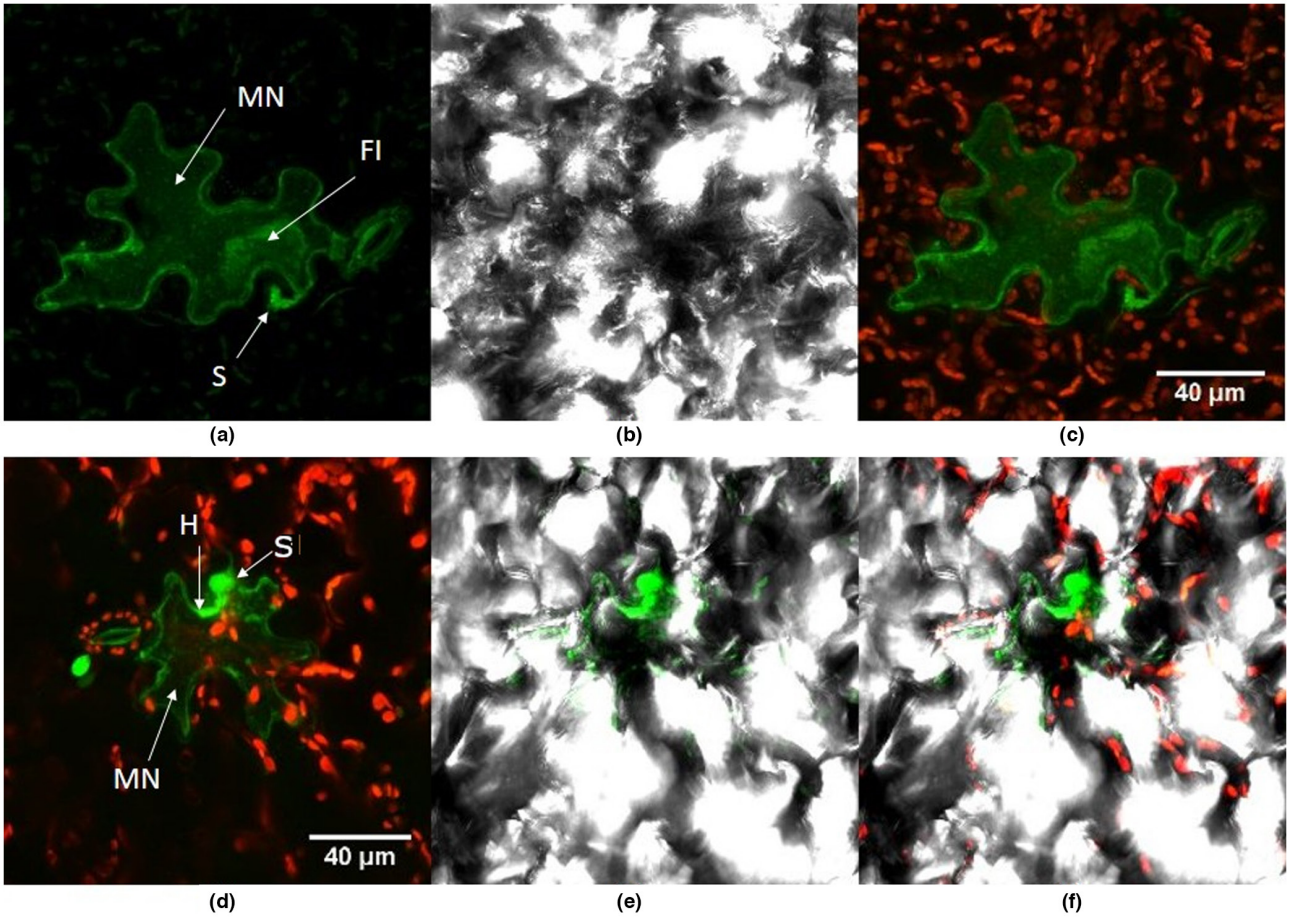
Visually healthy seedling lettuce leaves inoculated with GFP‐labelled *Botrytis cinerea* Bcgfp1‐3, 5 days after inoculation at the two‐leaf stage, 7 days after sowing, and viewed using confocal laser scanning microscopy. (a) Superimposed images from successive focal planes (stacked *z*‐sections) from the green channel, showing an autofluorescing cell (MN) with fungal inclusion (FI) and spore (S). (b) Bright‐field illumination of (a). (c) Superimposed images from the red and green channels showing reduced density of chloroplasts in the autofluorescing cells. (d–f) Within the same field of view, (d) shows *z*‐stacked red and green channels, (e) shows green and bright‐field, and (f) shows red, green and bright‐field of *B*. *cinerea* hyphae (H), an autofluorescing stoma and an autofluorescing cell (probably a microscopic necrotic lesion, MN) with fungal inclusion (FI).

By 5 days after inoculation symptomless lettuce leaf tissues often had green autofluorescent epidermal cells and guard cells (Figure 3a–f). The walls of these cells fluoresced brightly and were sometimes hard to distinguish from fungal hyphae growing closely associated with the cell wall. The autofluorescent cells had fewer chloroplasts than adjacent cells (Figure 3c,d,f) and were always associated with ungerminated or germinated spores, suggesting the cells were single cell necrotic lesions. Under bright field illumination the cells were apparently damaged (Figure 3b,e). Most had a distinguishable, green fluorescent, thick, fungal inclusion inside the plant cell, often closely associated with the plant cell wall, with definite margins but a variable shape (Figure 3a,d). These microscopic lesions were always restricted to one or a few plant cells, regardless of the age of the sample. Germinating spores sometimes produced more than one germ tube. The germ tubes or hyphae ran along the plant cell wall.

##### 10 dpi

**FIGURE 4:**
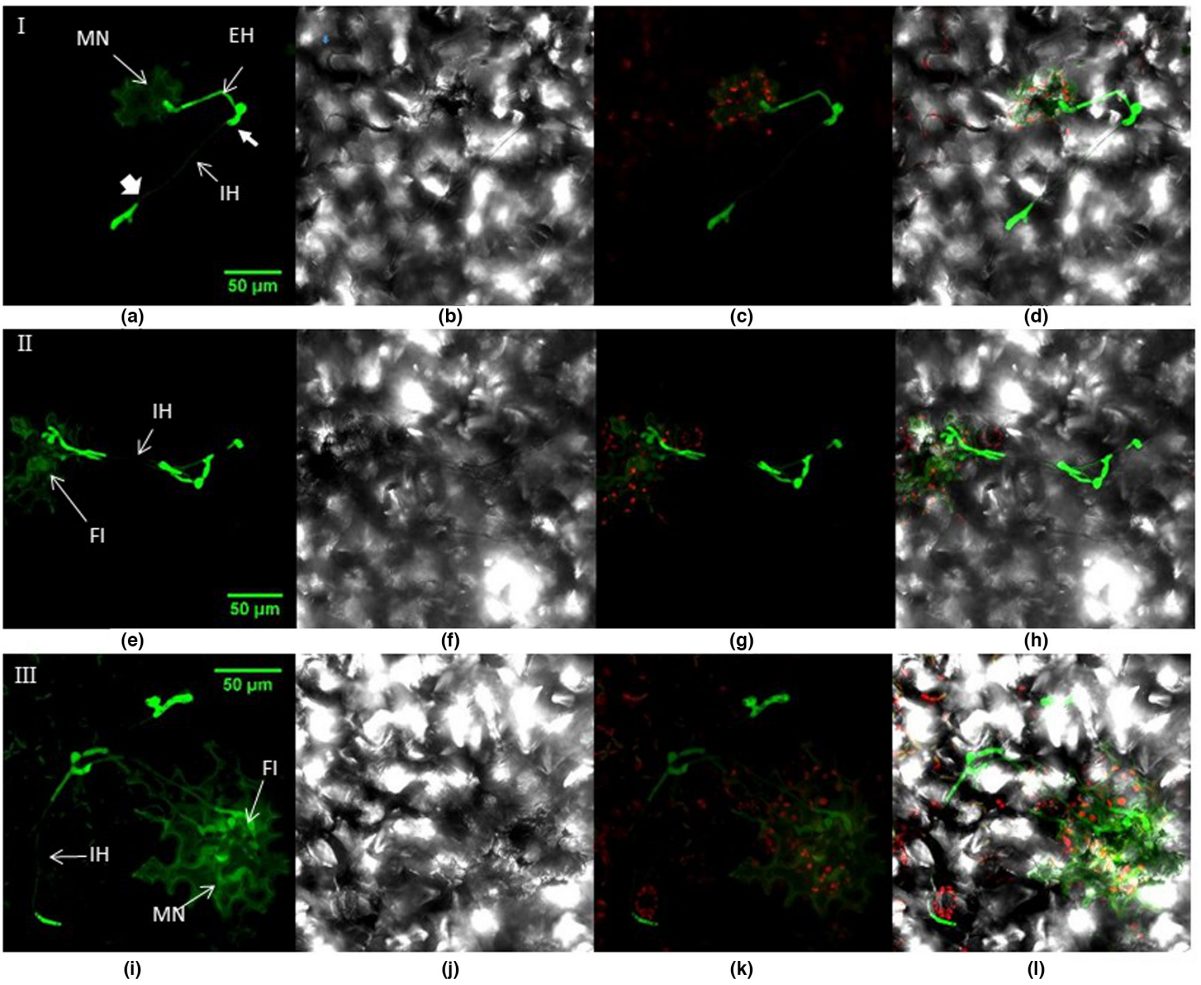
Seedling lettuce leaf inoculated with GFP‐labelled *Botrytis cinerea* Bcgfp1‐3, 10 days after inoculation, viewed by confocal laser scanning microscopy. The superimposed images from successive focal planes (*z*‐stack) in the green and red channels and bright‐field images show fungal hyphae developing from microscopic necrotic lesions in leaf samples. The images in rows I‐III are from three different samples. (a) *Z*‐stacked images in the green channel show a single necrotic cell (MN) associated with a hypha (EH), which has grown on the external leaf surface before entering the cuticle and growing inside it or between epidermal cells (IH); (b) bright‐field image of (a); (c) red and green channels of (a); (d) merged image of (a–c). (e–h) Another sample showing similar structures to (a–d), viewed with (e) green channel, (f) bright‐field, (g) red and green channels and (e) bright‐field, red and green channels. Prominent fungal inclusions (FI) are visible with attached hyphae growing within and on the surface of the leaf. (i–k) A third sample, viewed with channels as for (a–d), showing a multicellular microscopic lesion (MN) autofluorescing around a fungal inclusion.

**FIGURE 5:**
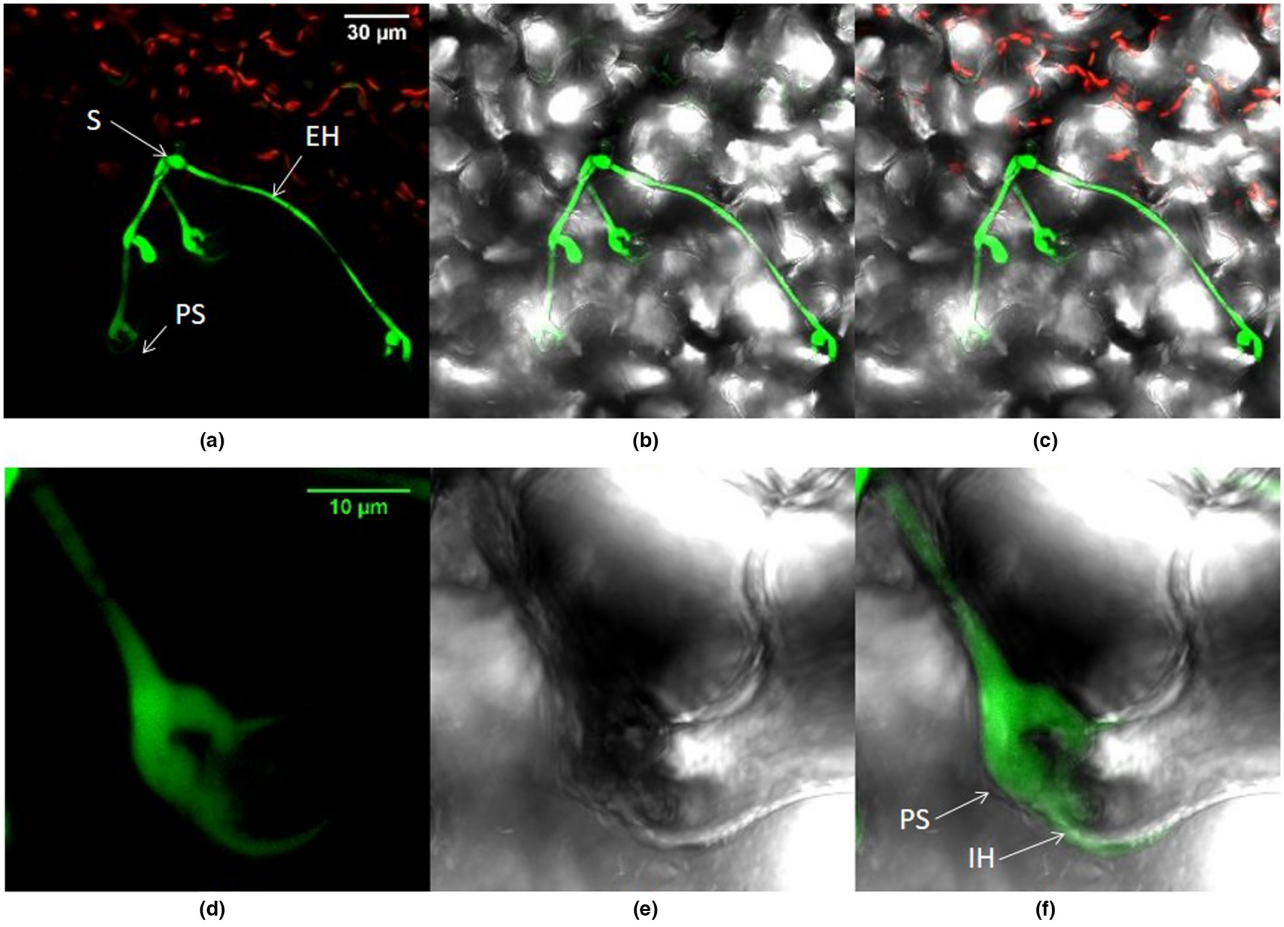
Lettuce leaf inoculated with GFP‐labelled *Botrytis cinerea* bcgfp1‐3, 10 days after inoculation, viewed by confocal laser scanning microscopy. (a–c) A conidium (S) has produced hyphae that have grown externally, on the leaf surface (EH), and internally (IH) with penetration structures (PS). (a) Superimposed images from successive focal planes (*z*‐stacked) viewed with the green and red channels. (b) Image area as (a) with green channel superimposed on bright‐field. (c) Image area as (b) with red channel also. (d–e) Part of the image area in (a) viewed at higher magnification, showing a finger like, Y‐shaped penetration structure (PS) and hypha leading to it from the cuticle (IH). (d) *Z*‐stacked image viewed with green channel; (e) image viewed in bright field showing epidermal cell boundaries; (f) superimposition of (d) and (e).

The fungal inclusions present in the microscopic necrotic lesions produced hyphae that spread within and on the healthy leaf tissues as branched mycelium (Figure 4). By this time not all mycelial structures could be unequivocally attributed to a particular spore and the pattern in which mycelial structures had developed was not always obvious. Portions of mycelium that grew inside the tissue usually appeared from the surface to be thinner than the portion growing outside (Figure 4a,e,i and associated multichannel superimpositions). In some sites of infection the internal mycelium was weakly detected (Figure 4, ‘IH’), but became much brighter when hyphae appeared on the outer leaf surface (Figure 4, bold arrows). The terminal ends of hyphae mostly produced broad or enlarged structures below the cuticle, often branched. These terminal structures penetrated the plant tissues at cell junctions (Figure 5c,f).

##### 12–15 dpi

**FIGURE 6:**
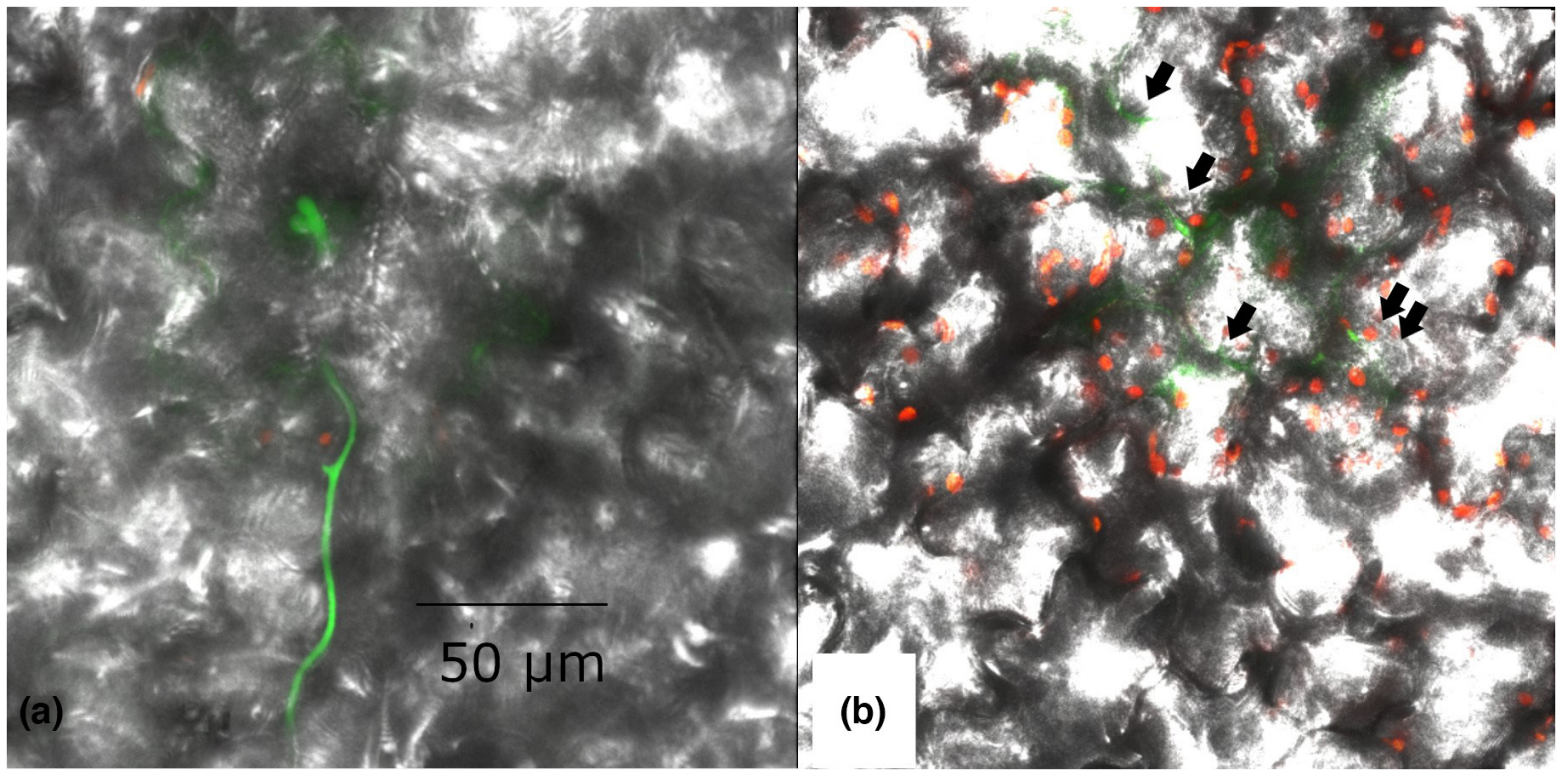
Growth of GFP‐labelled *Botrytis cinerea* Bcgfp1‐3 in lettuce 12 days after inoculation using confocal laser scanning microscopy. Images show the same section at two focal planes, with (a) showing focus at the epidermis and (b) focus in the mesophyll below it. (a) Mycelium growing on the leaf surface; (b) mesophyll cells with intact chloroplasts, extensive voids, and mycelium growing through it, away from the surface mycelium and mostly not parallel to the section, lying just outside the focal plane. Arrows point to some areas of interior mycelium.

**FIGURE 7:**
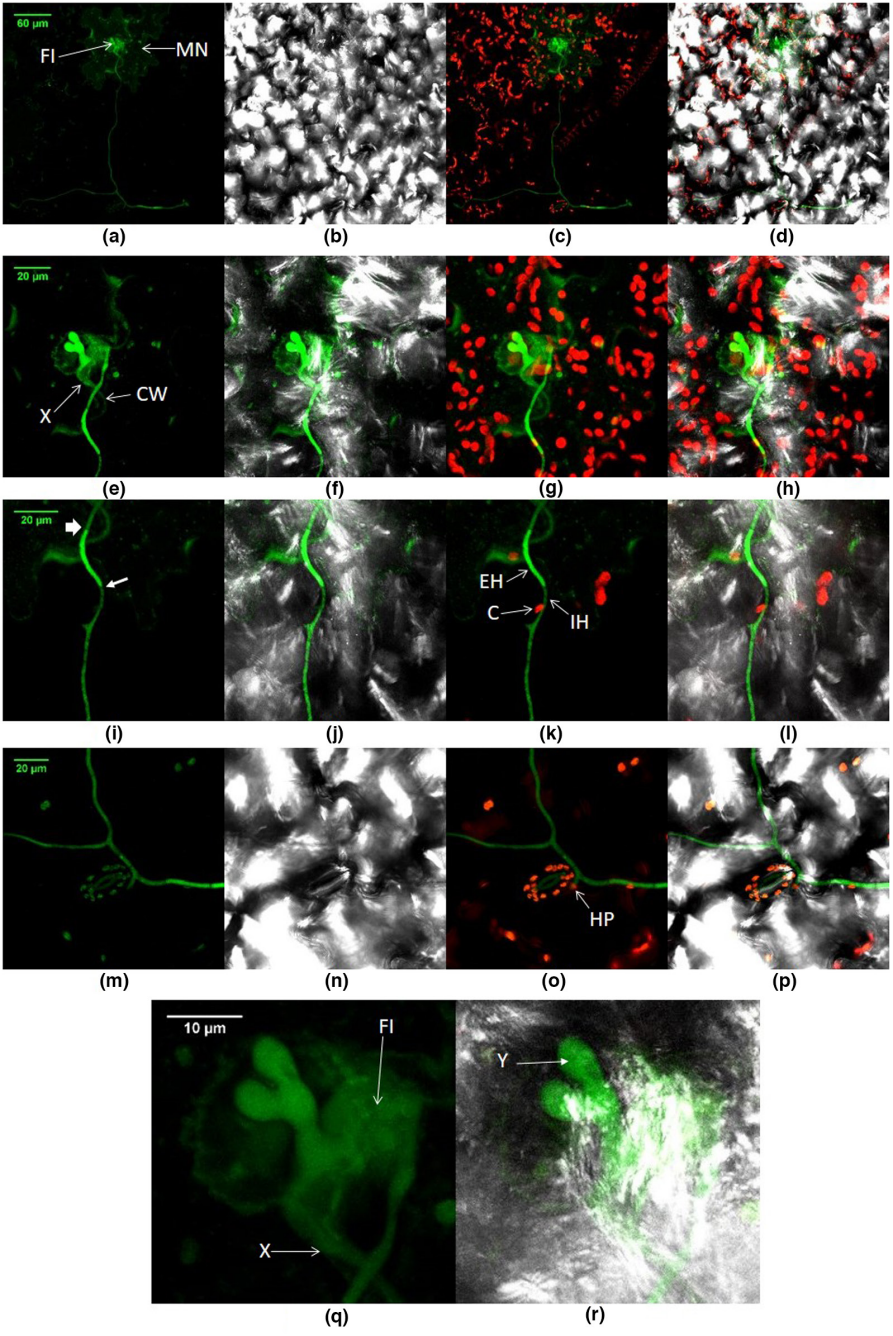
Lettuce leaf containing GFP‐labelled *Botrytis cinerea* Bcgfp1‐3, 12 days after inoculation. Detailed view of a microscopic necrotic lesion (MN), fungal inclusion and spreading hyphae in a lettuce leaf sample that had symptomless infection. (a, e, i, m, q) Superimposed images from successive focal planes (stacked *z*‐section) from green channel; (b, f, j, n, r) image area as (a, e, i, m, q) superimposed on bright‐field image; (c, g, k, o), image area as (a, e, i, m, q) with stacked *z*‐sections from red and green channels; (d, h, i, p) image areas as (a–i) with stacked green and red *z*‐sections superimposed on bright‐field. (a–d) Fungal spread from an initial site of origin (MN). The fungal inclusion (FI) has produced a hypha about 500 μm long before branching into two portions each about 250 μm at approximate right angles to the primary hypha. (e–h) Higher magnification of the microscopic necrotic cell and initial orientation of hyphae in (a–d). (i–l) Detail of hyphal growth. Less distinct regions of the hypha show where it grows internally, confirmed by the presence of chloroplasts above it marked C. (k and l) The hypha grows internally (IH) and externally on the leaf tissue (EH). (m–p) Hyphal growth between and above epidermal cell walls; note location marked HP, where growth is clearly below the cell. (q and r) Detailed view of panels (e–f); damaged cell boundary is apparent, with a fungal inclusion present and partly following the plant cell wall, and a hypha (marked X) has grown along the cell wall. The inclusion is below the cell surface and is connected to an appendage‐like structure that is exposed on the outer surface (Y).

**FIGURE 8:**
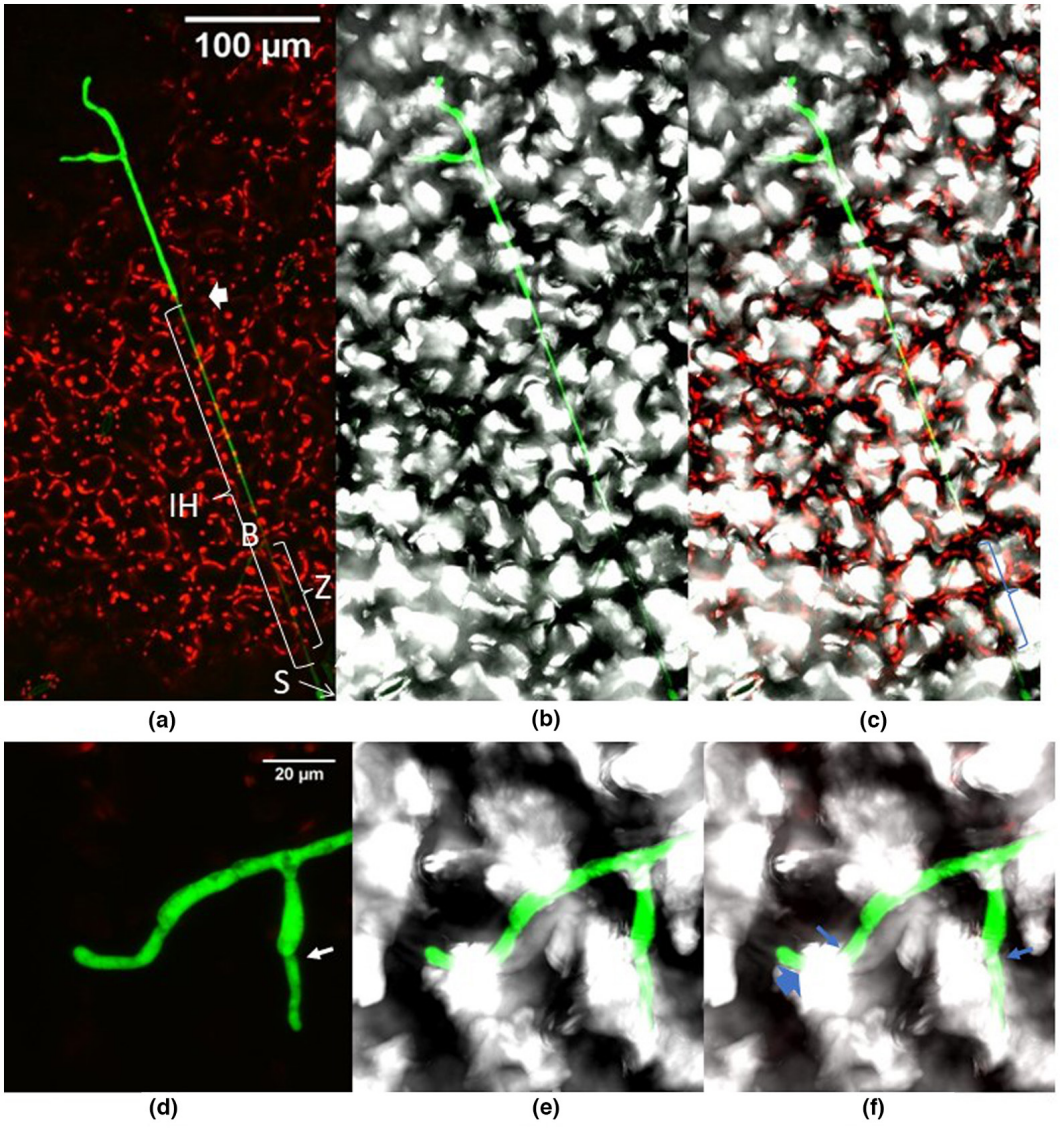
GFP‐labelled *Botrytis cinerea* Bcgfp1‐3 growing inside a symptomless lettuce leaf, viewed by confocal laser scanning microscopy 15 days after inoculation. (a and d) Superimposed images from successive focal planes (stacked *z*‐section) from green and red channels; (b and e) superimposed images from green channel and bright‐field; (c and f) superimposed images from green and red channels and bright‐field. (a–c) A conidium (S, based on shape and size) is connected to a long, straight, hypha. The main hypha and its branch (B) appear interior (IH) to chloroplasts, especially in the zone marked Z: compare (a) with (c). The arrow marks where hyphal morphology changes. (d–f) Detailed view of the terminal end of the mycelium; hyphae are intermittently obscured by epidermal cells or cuticle [arrows; compare (d) with (e) and (f)].

Fluorescence in internal focal planes (perpendicular to the leaf surface), in the mesophyll below the epidermal layers (Figure 6b), was detected well away (in the *x–y* plane, parallel to the leaf surface) from fluorescence detected on the surface (Figure 6a, 12 dpi; File S1). Hyphae were found below cells containing chloroplasts, therefore growing internally. However, the hyphae mainly grew along the cell junctions at the leaf surface, occasionally penetrating the epidermis to grow inside the leaf (Figure 7, 12 dpi; Figure 8, 15 dpi).

##### 30 dpi

**FIGURE 9:**
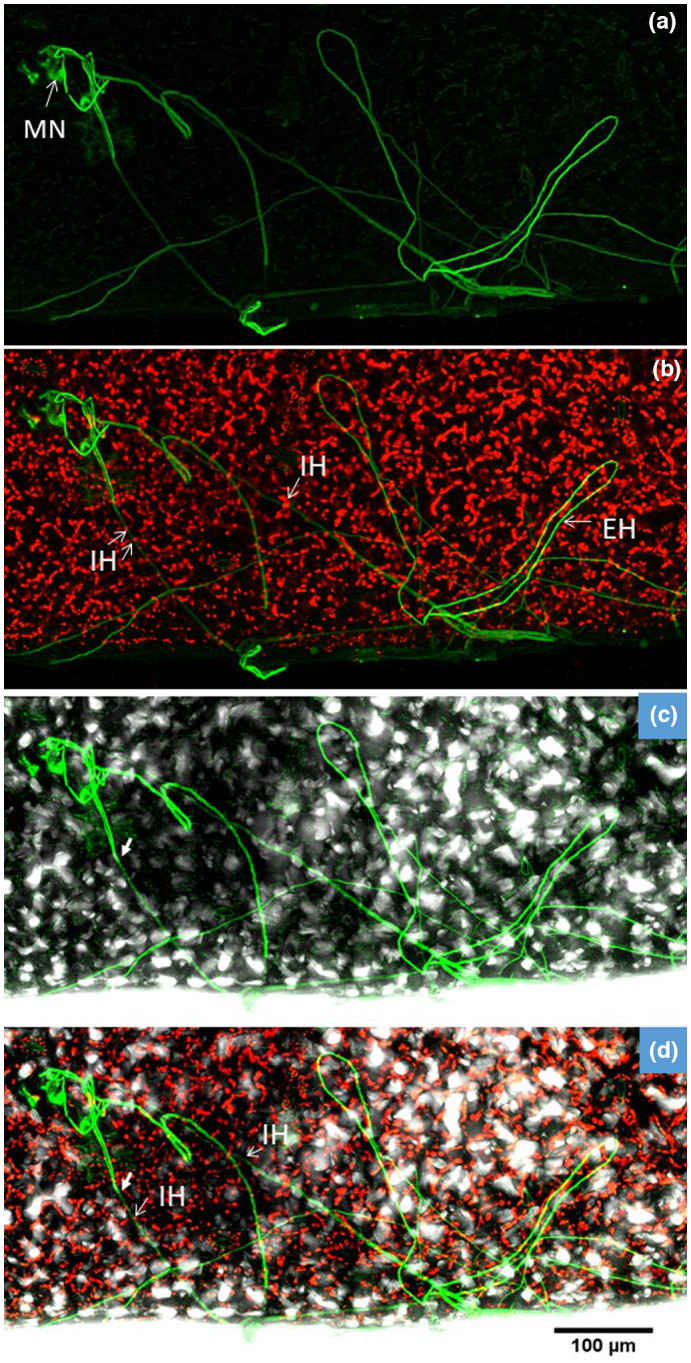
GFP‐labelled *Botrytis cinerea* Bcgfp1‐3 spreading in and on apparently healthy lettuce leaf tissue, 30 days after inoculation, viewed by confocal laser scanning microscopy. (a) Images from green channel, showing hyphae spreading from microscopic necrotic lesions (MN); (b) superimposed images from green and red channels, showing locations of hyphal growth below or within the chloroplast‐containing layers (IH) and hyphae on or above the external surface of the leaf (EH); (c) superimposed images from green channel and bright‐field; (d) superimposed images from red and green channels and bright‐field. The mycelium apparently originates from fungal inclusions in microscopic necrotic lesions and then spreads both outside the cuticle and inside the epidermis and cuticle of the leaf. Hyphae can be seen below the level of cells containing chloroplasts (b and d).

Long hyphae were observed near the leaf margin. They grew both inside and outside the leaf (Figure 9). They arose from microscopic necrotic lesions with fungal inclusions as at 5 dpi (Figure 9a,b). These fungal inclusions produced a number of hyphae and formed mycelium on and in the leaf. The inclusions were larger than in earlier samples; in the samples taken at the longest times after inoculation (>30 dpi) about 2/3 of an infected plant cell was filled by the inclusion (cf. Figures 3d and 9a). However, apart from these microscopic necrotic lesions all other plant cells in the leaf tissue remained apparently healthy (Figure 9c,d).

#### 3.2.2 Arabidopsis

##### 4–10 dpi

**FIGURE 10:**
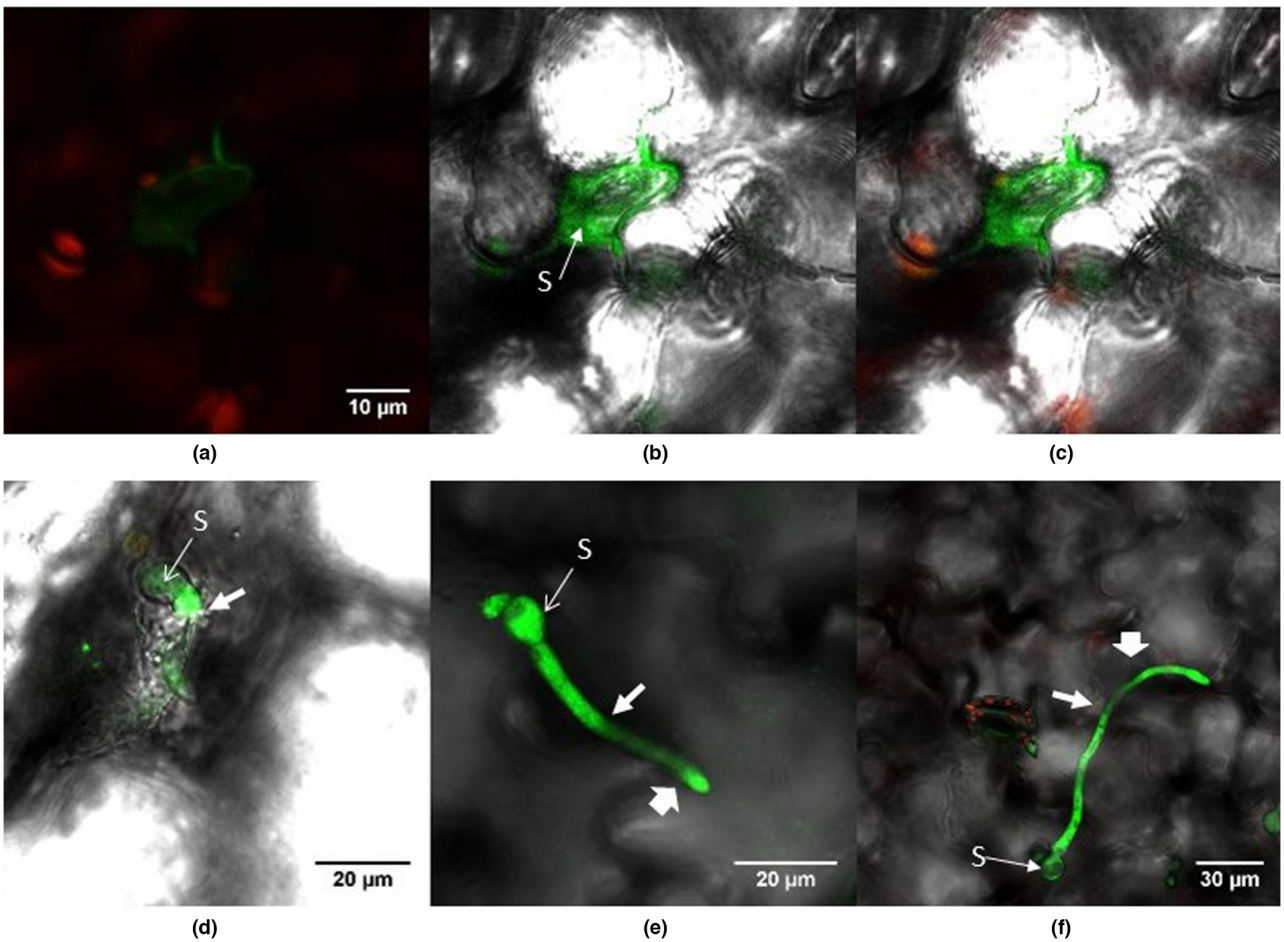
Spore germination and germ tube development by GFP‐labelled *Botrytis cinerea* Bcgfp1‐3 on *Arabidopsis thaliana* rosette leaves, viewed by confocal laser scanning microscopy. (a–c) A germinated spore (S) and its hypha, which has spread around a guard cell, 4 days after inoculation, viewed by superimposed images from red and green channels (a), images from green channel superimposed on bright‐field (b), and superimposed images from red and green channels and bright‐field (c). (d–f) Nine days after inoculation, images show germ tubes developing from a spore, which directly penetrate and grow inside the leaf tissue (d) or on the leaf surface (e and f), possibly with some interior growth starting where indicated by narrow arrow, followed by surface growth resumed at wide arrow.

**FIGURE 11:**
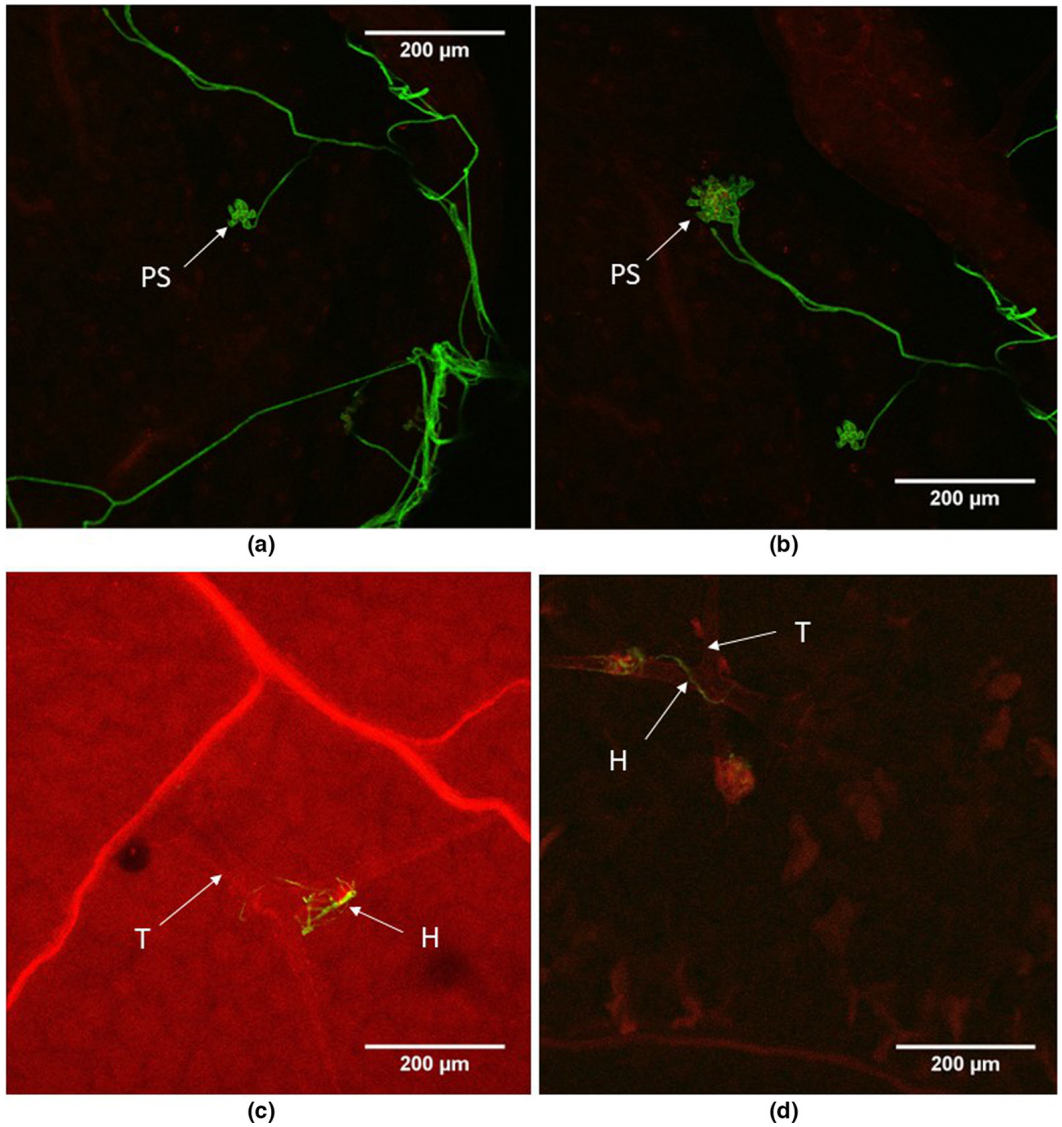
Penetration structures and external growth produced by *Botrytis cinerea* isolate B05.10 on *Arabidopsis thaliana* rosette leaves, 10 days after inoculation. Leaf samples were stained with WGA‐FITC and propidium iodide and viewed under a TCS SP2 confocal laser scanning microscope. The fungus produces a branched mycelial network near to the leaf margin (a and b). The terminal ends of some hyphae produce differentiated penetration structures (PS). Fungal mycelium was also frequent on the trichomes of the leaves (c and d). H, hypha; T, trichome.

*A*. *thaliana* had symptomless infections in experiments with both *B*. *cinerea* isolate B05.10 and its GFP‐labelled derivative Bcgfp1‐3. However, recovery of *B*. *cinerea* from surface‐disinfested tissue was sparse (Figure 1b). The fungus showed direct tissue penetration; germ tubes penetrated epidermal cells and guard cell walls. Microscopic necrotic lesions were detected more rarely in *A*. *thaliana* than in lettuce (Figure 10: a–c 4 dpi, d–f 9 dpi). Differentiated local structures presumed to be associated with fungal penetration of the leaf were common (Figure 11a–d: 10 dpi). The fungus also grew on the outer surface of root samples (File S2; 10 dpi), which was consistent with recovery of cultures from unsterilized but not from surface‐disinfested roots.

##### 15–20 dpi

**FIGURE 12:**
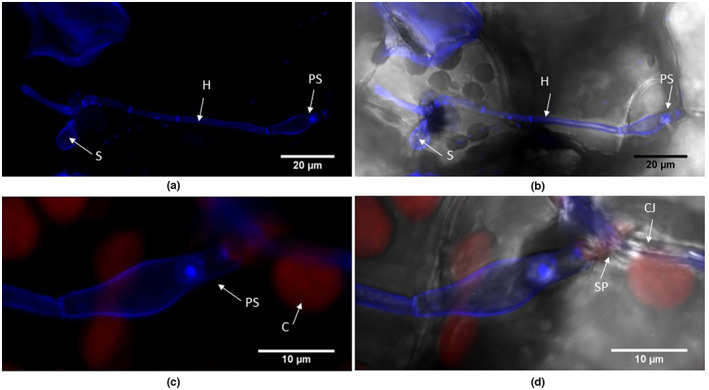
Penetration structures and external growth produced by *Botrytis cinerea* isolate B05.10 on *Arabidopsis thaliana* rosette leaves, 15 days after inoculation. The tissue sample was stained with calcofluor, counterstained with propidium iodide and viewed under a TCS SP5ii confocal laser scanning microscope. (a and b) Superimposed images from successive focal planes (*z*‐stack) of blue channel (a) and the same image superimposed on bright‐field (b), showing a germinated spore (S) that has produced two hyphae; the terminal end of one hypha (H) is modified as a penetration structure or infection peg (PS). (c and d) Right‐hand side of image in (a), viewed as a *z*‐stack of blue and red channels (c) and as superimposed images of the red and blue channels and bright‐field (d), showing the penetration structure, which is broader at the site of penetration (SP) below the adjacent chloroplast (C).

**FIGURE 13:**
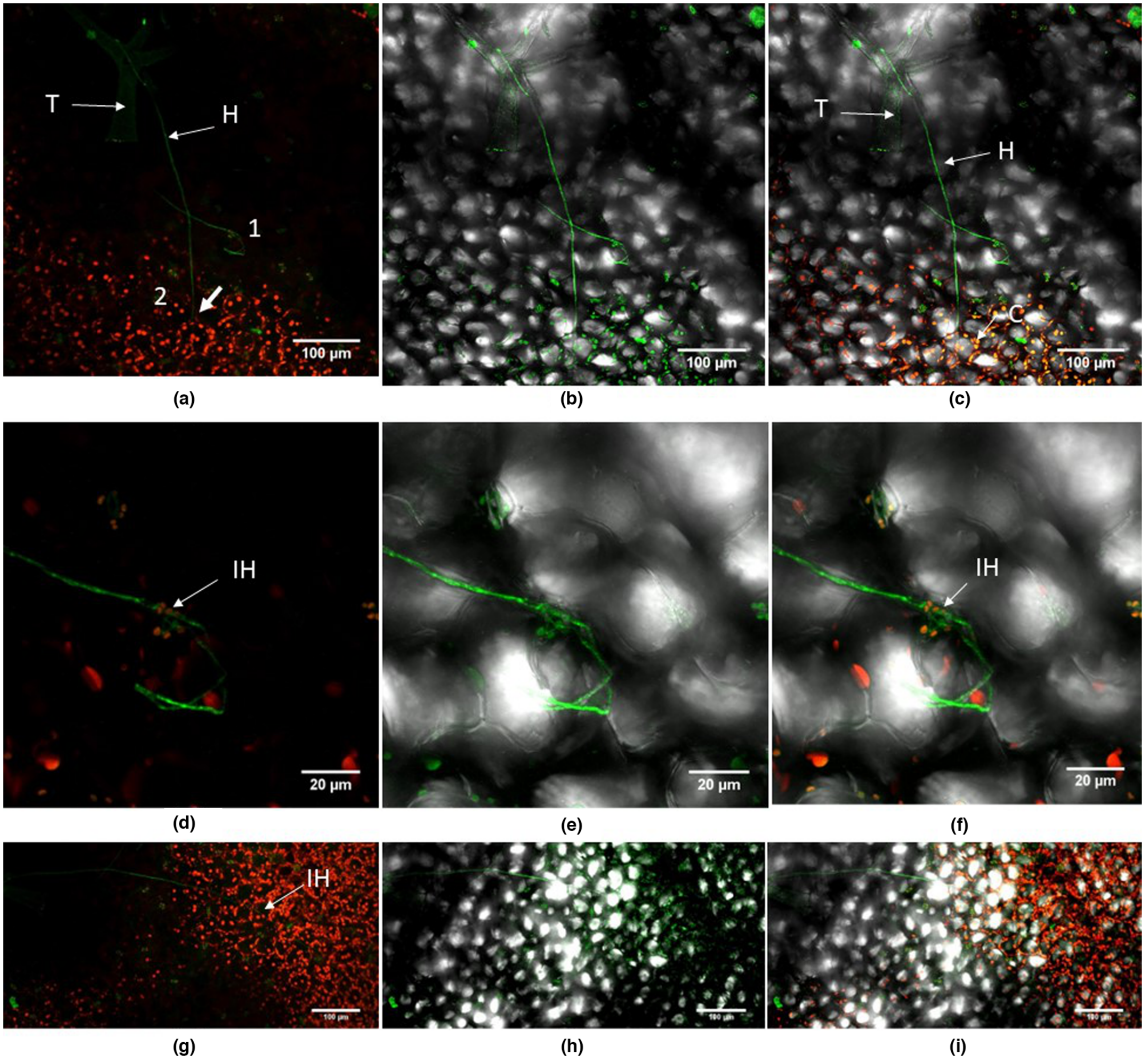
GFP‐labelled *Botrytis cinerea* Bcgfp1‐3 growing on a stem leaf of *Arabidopsis thaliana*, 20 days after inoculation. Each image is a combination of superimposed images from successive focal planes (stacked *z*‐sections) of different channels (red, blue and bright‐field) obtained by confocal laser scanning microscopy. (a, d, g) images from green and red channels, (b, e, h) images from green channel superimposed on bright‐field, and (c, f, i) images from red and green channels superimposed on bright‐field. (a–c) Fungal hyphae (H) spread from the trichome (T) to the leaf surface; 1 and 2 indicate the zones magnified in images (d–i). (d–f) A magnified portion of zone 1 in (a) where the end of the hypha penetrates one side of a guard cell wall (CW) and grows to other side. (g–i) Magnified images of zone 2 in (a), where a hypha (IH) originating from the trichome has penetrated the leaf and grown within the mesophyll below some chloroplasts.

As well as on leaves, GFP‐labelled *B*. *cinerea* was seen on stem and stem–leaves that had developed since inoculation (Figure 12, 15 dpi). The terminal end of some hyphae produced a swollen structure possibly similar to an infection cushion (a multicellular appressorium) (Figure 12d). On the leaf surface some hyphae ran extensively parallel to the surface (Figure 13d–f), with some evidence of internal growth (Figure 13d,f, 20 dpi). *B*. *cinerea* hyphae were often associated with trichomes in leaf, stem and stem–leaves (Figure 13a–c, 20 dpi); the conidia germinated on the trichome, the germ tube then curled round the trichome and moved down to the epidermis. In other observations a spore germinated at the base of the trichome and the germ tube climbed to the tip of the trichome. Hyphae also connected adjacent trichomes.

## 4 DISCUSSION

Earlier work with several host plants has shown that *Botrytis* species can spread extensively from the initially inoculated organs into newly produced host tissues before symptoms appear anywhere in the host (Barnes & Shaw, 2003; Emmanuel et al., 2018; van Kan et al., 2014; Shaw et al., 2016; Sowley et al., 2010). The present experiments were intended to observe directly asymptomatic growth of *B*. *cinerea* in *L*. *sativa* and *A*. *thaliana* and clarify its relationship to the host. Cultures and PCR were used to confirm the presence of the pathogen, and surface disinfestation demonstrated that some of the pathogen was growing in internal parts of *A*. *thaliana* as has been demonstrated previously for *L*. *sativa* (Emmanuel et al., 2018; Sowley et al., 2010). Lettuce and *A*. *thaliana* showed symptoms at about 3 months and 1 month after inoculation, respectively. During the intervening period, the fungus remained alive. However, in a leaf, out of hundreds of inoculated spores, typically fewer than 10 spores produced a long spreading mycelium. This mycelium spread from the site of inoculation to other parts of the plant. The observations reported here suggest that this spread was by both external and subcuticular growth, with occasional growth in internal regions of the host. Recovery of the pathogen from samples after surface disinfestation indicates that *B*. *cinerea* was growing inside the host, as disinfestation would kill exposed hyphae.

The depth of field of confocal laser scanning microscopes is less than the thickness of a lettuce leaf, so growth within the apoplast may have been underestimated. Nonetheless, the images show patterns of development consistent with the long distance spread within plants deduced from culture techniques, as well as some unexpected features.

Experiments with Bcgfp1‐3 were necessarily conducted in appropriate confinement, and experiments with unlabelled B05.10 were conducted with careful isolation procedures to avoid confusion with other fungi or with uncontrolled airborne inoculation. Light levels were therefore equivalent to strong shade in a greenhouse or the open. In previous work, low light levels have been shown to greatly reduce the proportions of lettuce plants and tissues that carry asymptomatic infection (Shaw et al., 2016). Therefore, some of the phenomena observed in the present study may have a different frequency or be atypical in some other way under brighter light conditions. In particular, in stronger light, internal growth of *B*. *cinerea* may go deeper into leaves and growth may be more extensive within vascular tissue; this would lead to more frequent recovery of *B*. *cinerea* from disinfested tissues from plants grown in bright light.

Mycelium was not seen inside root tissue of *Arabidopsis*. This is consistent with rare recovery of *Botrytis* by culturing from surface‐disinfested roots of *Arabidopsis*, in contrast to the very frequent recovery seen in *Taraxacum* (Shaw et al., 2016) and *L*. *sativa* (Sowley et al., 2010). External root infestation in *Arabidopsis* could arise from spread of mycelium from aerial shoot structures but could equally arise from mycelia developing from spores deposited on the soil during inoculation.

The fungus *B*. *cinerea* is generally considered to be a necrotrophic pathogen. Previous studies have provided evidence that the mode of inoculation influences the mode of interaction with the host and subsequent symptom expression (Emmanuel et al., 2018; Holz et al., 2007; Shaw et al., 2016). For laboratory experiments, inoculations often use droplets containing hundreds of spores in nutrient media (Choquer et al., 2021; Holz et al., 2007). This high spore density concentrates infection of the plant to a small area in direct contact with the droplet, underlying cells are killed, and the fungus establishes a primary lesion. When conditions favour further disease development, the fungus starts a vigorous outgrowth, resulting in rapid maceration of plant tissue, on which the fungus sporulates (van Kan, 2005). In the present experiments, lettuce and *A*. *thaliana* were inoculated with relatively sparse spore dusting, without initial surface wetness or nutrients. Spores of the same isolates suspended in droplets of potato dextrose broth produced visible necrotic lesions on both species within 48 h and conidiophores in 3–4 days, but spore dust inoculation took several weeks to show visible symptoms. In both host species, with dust inoculation, single dead epidermal cells were seen that seemed to form the origin of spreading mycelial networks, without further necrosis.

Swelling of tips was sometimes apparent on the germ tube or the hyphae growing on the outer surface of leaves. These broad swollen tips mostly penetrated the cuticle layer, usually at a cell junction, and lay inside the tissue, below the level of the rest of the hypha; this suggests an appressorial‐like function. *B*. *cinerea* has been reported to produce appressoria‐like structures of two types during penetration of plant tissue: swollen “Y” shapes, as seen in Figure 5, and clumps of small cells, which we did not observe. In contrast, Choquer et al. (2021) found single cell appressoria on several hosts. However, these were seen following inoculation of 10^3^ conidia in single droplets of nutrient medium. The Y‐shape structures are not well differentiated (Tenberge, 2007) but appear in hosts across a wide taxonomic and anatomical range (Garcia‐Arenal & Sagasta, 1980; Zhang et al., 2010). “Multifinger” swellings at hyphal tips have also been observed in *B*. *cinerea* strains expressing β‐glucuronidase constructs, made by the same approach as Bcgfp1‐3, but inoculated in droplets under conditions that did not favour spreading lesions (Stefanato et al., 2009). Similarly, in the infection of bean (*Vicia faba*) leaves by dry conidia of *Botrytis fabae*, Cole et al. (1996) found a distinct pad of amorphous material that encircled the short germ tube at the site of penetration. They suggested that this matrix material acts as an adhesive pad and thus serves to secure the position of the germ tube at the site of penetration. (However, the relevance to *B*. *cinerea* is uncertain because *B*. *fabae* is specialized to *Vicia faba* and does not seem to proliferate extensively without causing necrotic symptoms).

The infection processes inferred in this experiment are similar to infection processes in some other plant‐pathogenic fungi. Several fungi in the Sclerotiniaceae family can cause symptomless infection (Andrew et al., 2012); however, extensive spread over newly grown host tissues has not been not widely reported. In *A*. *thaliana*, we observed that *B*. *cinerea* grew predominantly on the outer surface of the plant and, in this way, became associated with new plant growth. By contrast, in lettuce, we observed spread of *B*. *cinerea* to large areas of the leaf. Hyphae grew on the outer surface, then penetrated the cuticle, after which substantial lengths grew inside the tissue, with some differences in appearance from the surface growth. The hyphae of *B*. *cinerea* are septate, allowing differentiation in appearance and function between cells or zones, as observed in the related fungus *Sclerotinia sclerotiorum* (Peyraud et al., 2019).

Fungal mycelium was frequently detected in *A*. *thaliana* on the trichomes of rosette leaves, and also observed on the stem, stem–leaves and plant organs that developed several days after inoculation. This suggests that growth beneath the cuticle or epidermis acts as a refuge if tissues are surface disinfested, although the bulk of growth through the developing host may be on the surface (especially in *A*. *thaliana*).

Deposition of callose on the cell wall has been reported in many plant–pathogen interactions, particularly during the defence reaction of hosts tolerant to fungal pathogens (Wang et al., 2021). In the present work, plant cell wall thickening and autofluorescence of cell walls was noted at the site of infection, most prominently in lettuce; in *A*. *thaliana* it was restricted to one or two cells. Some of these autofluorescing cells contained fungal inclusions, and fungal mycelium arising from these cells spread to other areas on the leaf surface, indicating that these cell wall changes must serve as a quantitative rather than qualitative barrier to pathogen growth.

Fluorescent compounds accumulate as part of defence responses to many pathogens in lettuce. The amount and location of the production of the fluorescent plant defence compound lettucenin A affects the ability of *Botrytis* to progress to full virulence upon initial penetration of the leaf (Bennett et al., 1994). When *Botrytis* was inoculated under disease‐favouring conditions (mycelium or spores in nutrient‐rich liquid inoculum), the ratio of lettucenin A to inoculum was too low to prevent spreading lesions; however, inoculation in pure water led to locally high concentrations of lettucenin A, sufficient to contain the fungus upon initial penetration (Bennett et al., 1994). Similarly, in our study, the penetration by single germ tubes from a single dry spore could trigger the production of sufficient defence compounds (potentially including the fluorescent lettucenin A) to contain the pathogen within the microscopic lesions observed from 5 dpi onwards. The fine balance between production of defence compounds and infection with *B*. *cinerea* has also been demonstrated in *Nicotiana tabacum*. On this host, the interaction could be shifted towards increased virulence by altering the inoculum from spores to mycelium (El Oirdi et al., 2010), or towards containment by increasing local resistance through transformation with a stress suppressing gene (Rossi et al., 2017). Some of the structures we have described around 10–12 dpi on *L*. *sativa* and *A*. *thaliana* (e.g., multipronged penetration structures, single fluorescent cells) were also observed by Rossi et al. (2017) at 3 dpi under unfavourable infection conditions on tobacco. However, those authors did not describe further growth, either sub‐ or extracuticular.

Droplet inoculation of high concentrations of *B*. *cinerea* conidia on *A*. *thaliana* under high humidity resulted in a rapid penetration, subcuticular growth and a majority of spreading lesions (e.g., van Kan et al., 2014; Shaw et al., 2016; Stefanato et al., 2009). However, various mutations in multiple loci render *B*. *cinerea* less virulent (Caseys et al., 2021). Specifically, Stefanato et al. (2009) reported mutant genotypes whose hyphae, despite germinating normally, remained mainly superficial and made structures at 1–2 dpi (Stefanato et al., 2009) that are similar to those observed in the present study at 10–12 dpi. High inoculum pressure using droplet inoculation with a weak pathogen resulted in a strong response accompanied by defence gene induction, defence compound production and extensive cell death (Kliebenstein et al., 2005; Stefanato et al., 2009). By contrast, we hypothesize that under the conditions of the current study, a single spore provokes a very localized host response upon cell penetration, weak enough to allow some intracellular subcuticular growth but just sufficient to contain the fungus within one or a few affected cells at the penetration site. A parallel case has been observed in potato inoculated with *Phytophthora infestans* (Vleeshouwers et al., 2000), where single epidermal cells undergo what appears to be hypersensitive cell death, but the pathogen emerges from them and still colonizes the potato leaf. In both cases, a plant response usually characterized as absolute is evidently quantitative.

The interaction between any particular *B*. *cinerea* strain and potential host plants is complex and depends on multiple host and pathogen factors (Corwin et al., 2016). As shown previously, potentially pathogenic strains can cause symptomless infections that have been likened to endophytic growth (Bastías et al., 2021; Shaw et al., 2016). The present work suggests that *B*. *cinerea* only grows subcuticularly in small stretches and, by elongating external hyphae away from microscopic necrotic lesions, avoids responses that would kill the whole mycelium.

Despite the absence of clear gene‐for‐gene interactions, *B*. *cinerea* is considered to encounter multiple induced defence reactions (van Kan, 2005). Upon droplet inoculation, the large biomass of *B*. *cinerea* is more likely to induce drastic host responses that might be co‐opted by the necrotrophic *B*. *cinerea* to induce cell death (Govrin & Levine, 2002). The balance of defence responses is probably dependent on the form and quantity of the inoculum. As argued above, a single spore from our dry inoculation produces single cell lesions from which mycelium can emerge. The observed differences in development could be due to differences in biomass, to adaptive gene expression patterns dependent on the inoculation method, or a combination of factors. Transcription of pathogenesis‐related *B*. *cinerea* genes in symptomless infections follows patterns distinct from those in actively necrotic lesions (Emmanuel et al., 2018), and may reflect diverse mechanisms for asymptomatic growth in diverse hosts. For example, in *L*. *sativa*, several pathogenicity‐related genes, including *Bcnepl* and *Bcbot1*, are very weakly expressed during the entire symptomless growth period. By contrast, in *A*. *thaliana*, these two genes are similarly expressed in symptomless stems at flowering 10 days after inoculation and in spreading necrotic infections of detached leaves 2 days after inoculation (Emmanuel et al., 2018).

In summary, the present study confirms previous studies showing that *B*. *cinerea* (and presumably related, epidemiologically similar species) can be present, without symptoms, in and on newly growing tissues in some hosts (Shaw et al., 2016). Hyphae spread both over the external surfaces of host tissue, and internally. The internal infections may be intermittent incursions from the host surface, or more extensive growth between cells in the apoplast; these observations are consistent with infection surviving surface disinfestation being common in some hosts. Spread of mycelium into the mesophyll or vascular tissues was not seen. A caveat is that the experiments were conducted under low light conditions, which are not favourable to asymptomatic growth (Shaw et al., 2016).

## Supporting information


**File S1.** This video uses a succession of optical sections to generate a 3D image of the epidermis and upper mesophyll of a lettuce leaf infected with *Botrytis cinerea* bcgfp1. No visual symptoms were apparent. As the view rotates from the upper surface to a position inside the mesophyll looking outward (cf. Figure 5), note the hyphae (green) passing through the tissue, becoming visible among the chloroplasts (red) inside the leaf in a slightly different zone from the epidermal colonization.

**File S2.** *Botrytis cinerea* isolate B05.10 growing on the outer surface of *Arabidopsis thaliana* roots, 10 days after inoculation. Stacked optical sections. Root samples (a–d) were stained with WGA‐FITC (fluorescing green); plant cells were stained with propidium iodide (fluorescing red). The fungus (H) has grown as a long mycelium or mycelial network on the external surface of the root (R).

## Data Availability

Files containing images contained in this publication are available from M.W.S. or C.J.E. Following severe hardware failure and the current difficult conditions in Sri Lanka, most images in the original file format, in particular unmerged *z*‐stacks, are currently unavailable.
