# Supplementary figures and images for: Microscope studies of symptomless growth of Botrytis cinerea in Lactuca sativa and Arabidopsis thaliana
Source: Plant Pathol. 2022 Dec 8;72(3):564–81. doi: 10.1111/ppa.13683 (PMC10952648; doi:10.1111/ppa.13683)

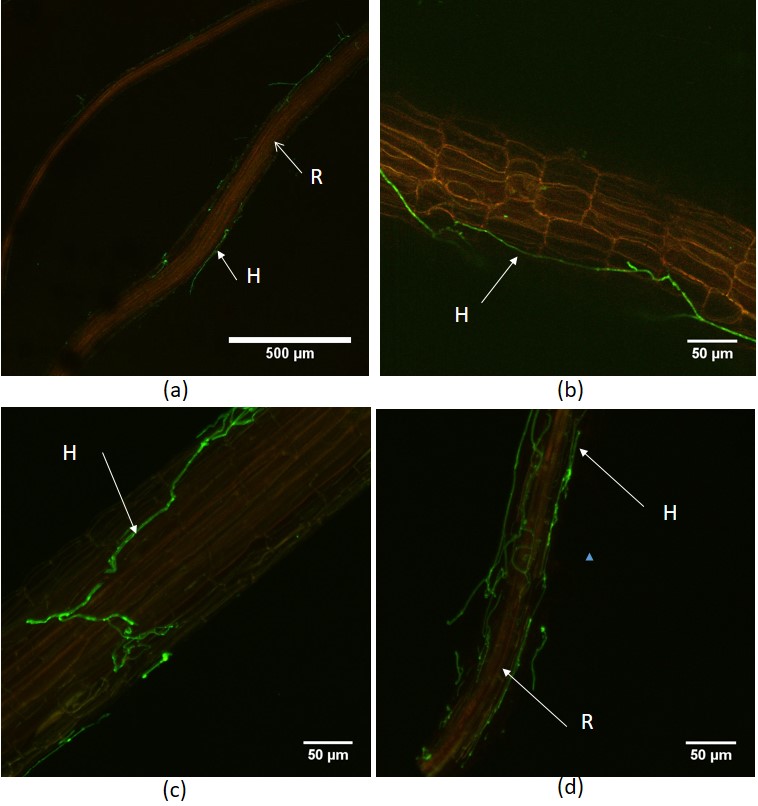

Supplement: Supplementary file 2 — File S2. Botrytis cinerea isolate B05.10 growing on the outer surface of Arabidopsis thaliana roots, 10 days after inoculation. Stacked optical sections. Root samples (a–d) were stained with WGA‐FITC (fluorescing green); plant cells were stained with propidium iodide (fluorescing red). The fungus (H) has grown as a long mycelium or mycelial network on the external surface of the root (R). [file PPA-72-564-s002.jpg]
